# Medical student-led implementation of preclinical abortion didactic session at a California medical school

**DOI:** 10.1186/s12909-023-04395-x

**Published:** 2023-06-14

**Authors:** Irene Masini, Katherine Rosecrance, Yamini Patibandla, Margot Barker, Anna Cardall Jarvis, Jasmine Patel

**Affiliations:** 1 School of Medicine, University of California, Irvine, United States; 2 Department of Obstetrics and Gynecology, University of California, Irvine, United States; 3 University of California, Irvine School of Medicine, Irvine, CA United States

**Keywords:** Peer education, Medical education, Abortion care, Pregnancy options counseling

## Abstract

**Background:**

Formal education surrounding abortion care during pre-clinical years of medical school is limited and will likely decrease with the overturning of Roe v. Wade. This study describes and evaluates the impact of an original abortion didactic session implemented during the pre-clinical years of medical school.

**Methods:**

We implemented a didactic session at the University of California Irvine outlining abortion epidemiology, pregnancy options counseling, standard abortion care, and the current legislative landscape surrounding abortion. The preclinical session also included an interactive, small group case-based discussion. Pre-session and post-session surveys were obtained to evaluate changes in participants’ knowledge and attitudes and to collect feedback for future sessions.

**Results:**

92 matched pre- and post-session surveys were completed and analyzed (response rate 77%). The majority of the respondents identified themselves as more “pro-choice” compared to “pro-life” on the pre-session survey. Results reflected significantly increased comfort discussing abortion care and significantly increased knowledge about abortion prevalence and techniques after the session. Qualitative feedback was overwhelmingly positive and reflected participants’ appreciation for the focus on the medical aspects of abortion care as opposed to an ethical discussion.

**Conclusions:**

Abortion education targeted to preclinical medical students can be implemented effectively by a medical student cohort with institutional support.

**Supplementary Information:**

The online version contains supplementary material available at 10.1186/s12909-023-04395-x.

## Background

On June 24, 2022, the United States Supreme Court overturned the constitutional right to abortion previously established by the historic Roe v. Wade decision [1]. Following this recent ruling on Dobbs v. Jackson Women’s Health Organization, 26 states are predicted to ban abortions based on already passed trigger laws [2]. At the time of writing this manuscript, two months after the overturning of Roe, nine states have prohibited abortions at any gestational age and four states prohibited abortion at greater than six weeks gestational age [3]. Not only is patient access to abortion care now limited, but critical abortion training for medical doctors is also severely impacted. With Roe v. Wade overturned, 44% of obstetrics and gynecology residents will be trained in a state where abortion would be made illegal, and access to some level of abortion training for residents is predicted to decrease from 92 to 56% [4]. In fact, the new legal landscape has already proven to restrict abortion training in states with highly restrictive abortion bands [5]. Although the legislation does not directly ban abortion training, a ban on abortion limits the volume of abortions and the practice settings in which they occur, thereby limiting training opportunities for trainees in affected states. In addition, states may ban state employees from participating in abortion care as they have in the past, again limiting abortion care for learners in state institutions.

In addition to medical residency training being affected, education for medical students pursuing all specialties will be limited as well. Only 32% of medical schools offer formal lectures covering abortion and 45% include abortion in their clinical curriculum [6]. Informal exposure to abortion is largely limited to clinical rotations and is expected to significantly decrease with Roe v. Wade overturned as access to abortion is severely restricted and eliminated. Both medical students who consider themselves in support of and against abortion have been shown to support abortion education in medical school [7]. As students at University of California Irvine (UCI) School of Medicine, we sought to address the gaps in our own curriculum surrounding abortion care. Thus, we proposed a medical-student-led abortion lecture and case-based discussion to be given during medical students’ second year. The objective of this study was to evaluate the efficacy of this session for preclinical medical students.

## Methods

We created a working group of five second and third-year medical students and a faculty member from the Division of Family Planning at UCI School of Medicine. First, we conducted a needs assessment by surveying the preclinical curriculum and clinical didactics sessions. Search inquiry included abortion and family planning education. In addition, we asked five students from each year if they had received abortion education at any point in their training thus far. We also asked a sample group of faculty who were in charge of curriculum development if they had provided any formalized abortion teaching. Through this process, we found a paucity of formal abortion education in both the preclinical and clinical years. We found that abortion was only briefly mentioned during a general ethics and values clarification lecture during first year of medical school and a lecture about prostaglandin medications during second year. We then researched other medical schools’ abortion curricula in order to gather information on best practices for abortion care education. We created a brief outline of goals for a novel preclinical abortion lecture and proposed the new session to the medical school administration. We received support to present an initial optional session to first- and second-year medical students with the possibility of it becoming a required part of the preclinical curriculum in future years if it was well received.

Our primary goal was to create an innovative lecture with clinically relevant material and an emphasis on interactive learning to keep audience members engaged. Specifically, our learning objectives were: (1) inform students of the different methods of abortion and the clinical scenarios for use, (2) increase medical student comfort with discussion of abortion and pregnancy options counseling, and (3) allow students to apply this knowledge to a clinical case scenario with a young pregnant patient seeking counseling at different points in her journey for pregnancy options. With these goals in mind, we developed a 45-minute didactic followed by a 15-minute facilitator-led small group discussion designed around a patient scenario, (Supplemental Item 1).

Given constraints of the COVID-19 pandemic, the session was conducted as a virtual encounter on Zoom, a video-conferencing platform. The didactic session was presented by a Family Planning faculty member as well as the medical students who developed the session and lasted 45 min. Following the didactic, the “breakout room” feature on Zoom was utilized for 15-minute small-group discussions. Third- and fourth-year medical students, residents, and faculty served as small group discussion leaders for groups of five to ten learners. This allowed for a more comfortable setting for discussion of the case given the sensitive nature of the topic. The presentation was offered twice, once during spring 2021 and again the following fall of 2021. The session was optional in the spring and mandatory in the fall for second-year medical students who had not already attended. Pre- and post-session surveys were designed to evaluate the presentation and gather feedback (Supplement 2 and 3). Surveys were designed by the researchers and each included 15 items, taking about five minutes to complete. Surveys were voluntary and anonymous; they included demographic information questions, three knowledge assessment questions, and six Likert scale questions to rate student attitude and comfort regarding abortion care and counseling. Qualitative feedback was obtained using free response questions integrated in the post-session survey. A three-digit anonymous subject identifier was created to match pre- and post-session surveys; respondents were instructed to use the first letter of their last name and their date of birth (e.g. D01 for John Doe born on 01/01/1990). Only matched subjects were included in the analysis. The surveys were developed and distributed to participants using the online platform REDCap. Stata Statistical Software: Release 16 (StatCorp. College Station, TX) was used to describe our variables. We compared matched data using McNemar’s test for discordant pairs.

## Results

Including both spring and fall iterations, 92 matched pre- and post-session surveys were completed out of 120 attendees for a response rate of 77%. Of the 92 students who completed the survey, 88 (96%) provided qualitative feedback. In our sample of participants, the average age was 26 years old (SD 2.3); 57% identified as female, and most reported White (41%) or Asian race (38%). On the pre-session survey, participants selected an average score of 80 from a visual analog sliding scale of 0 to 100 measuring personal beliefs on abortion with 0 representing a “pro-life” stance and 100 representing “pro-choice” (Table 1). Respondents were blinded to the numerical scale in order to avoid bias in answer choices. Two of the three knowledge questions showed significant increase in correct answers in the post-session survey (Table 2). After the presentation, more students correctly identified the percent of all US pregnancies that end in abortion (OR 13.3, p < 0.001) and the different methods of abortion (OR 48.0, p < 0.001). No significant increase was noted in correctly identifying the safety of abortion versus delivering at term (OR 0.64, p = 0.48); 86% of respondents answered this question correctly on the pre-session survey, compared to 90% on the post-session survey.

**Table 1:** Student Characteristics (n = 92)

Characteristic	Mean (SD) or N(%)
Age* (years)	26 (2)
Self-identified gender	
Male	40 (44%)
Female	52 (57%)
Nonbinary, Not listed	0 (0%)
Race**	
White	38 (41%)
Asian	35 (38%)
Black	10 (11%)
Middle Eastern	8 (9%)
Native Hawaiian or Pacific Islander	1 (1%)
Other	9 (10%)
Hispanic/Latino ethnicity	13 (14%)
Personal beliefs on abortion from scale of 0 (“pro-life”) to 100 (“pro-choice”)	80 (30)

*Age category (n = 90), **Other included American Indian, Alaska Native, or Not listed

**Table 2:** Comparison of Pre and Post Session Survey Responses (n = 92)

**Knowledge Question**	Pre-Session Survey correct response N(%)	Post-Session Survey correct response N(%)	Odds Ratio	95% Confidence Interval
In the US, about what percentage of all pregnancies end in abortion?	26 (28)	63 (69)	13.3	4.2–67.4
In the US, having an abortion is more dangerous to the pregnant person than carrying the pregnancy to term.	79 (86)	83 (90)	0.64	0.2–1.8
All of the following are methods of abortion except: *Medication abortion* *Vacuum aspiration* *Endometrial ablation* *Dilation & evacuation* *Induction of labor*	27 (29)	74 (80)	48.0	8.2–1934.9
**Attitude of Abortion Care and Education**	Pre-Session Survey: “Strongly Agree” and “Somewhat Agree” N(%)	Post-Session Survey: “Strongly Agree” and “Somewhat Agree” N(%)	Odds Ratio	Confidence Interval
*I will encounter patients who have had or are considering having an abortion*	88 (96%)	89 (97%)	-	-
*I feel comfortable talking to my patients about abortion options*	47 (51%)	85 (92%)	39.4	6.6–1579
*It’s important to me that I am knowledgeable about abortion options*	88 (96%)	89 (97%)	0	0–39
*I believe that all physicians should be knowledgeable about abortion options*	86 (94%)	87 (95%)	2	0.1–118.0
*It is part of a physician’s duty to provide comprehensive and accurate information about abortion to patients seeking this information*	85 (92%)	89 (97%)	0.2	0.004-1.8
**Post-session reflection**				
Did today’s session change your views or attitude regarding abortion?*	--	28 (30%)	--	
Will today’s session affect your future clinical practice?*	--	81 (89%)	--	

* Percent answered yes

Significantly more students responded “strongly agree” and “somewhat agree” on the post-session survey in response to the statement “*I feel comfortable talking to patients about abortion and pregnancy options”* (OR 39.4, p < 0.001) (Table 2). 89.0% answered “yes” to the question “*Will today’s session affect your future clinical practice?”*. Nearly one-third (30.4%) of students responded “yes” when asked, *“Did today’s session change your views or attitudes regarding abortion?”.* Of the participants who said their views had changed, free responses reflected that for many it was due to learning about the safety and prevalence of abortion. Of the participants who said it did not change their views, most mentioned their pre-existing support and knowledge surrounding this subject. One participant mentioned that their views were not changed because they were “influenced by religious and moral beliefs”. Another student praised the “emphasis on the patient-centered purpose of holding this informational session for individuals who might not personally be in favor of abortion but need to hear this material to help patients”. We obtained positive qualitative feedback from participants with appreciation of the unbiased approach and focus on the medical aspects of abortion care. Participants affirmed that “breakout rooms were particularly beneficial” and asked for more “time to complete questions” and “smaller breakout groups”. Students also emphasized the appropriateness and organization of the presentation (Table 3). Feedback after the first session for increased duration and smaller discussion groups were successfully addressed in the second session in the Fall.

**Table 3:** Selected quotes from students from post-session survey

Did today’s session change your views or attitudes regarding abortion? Describe why or why not.
*“I felt that depending on specialty, some providers may not need to know about options regarding abortion. But coming out of the session I feel that information is vital to learning medicine.”*
*“I realized I had many misconception about abortion regarding its safety and ethics.”*
*“My views on abortion are influenced by my religious and moral beliefs, neither of which were addressed in the talk today. That being said the autonomy of the patient is most important and I would never impose my beliefs on to them.”*
*“The most surprising evidence that was provided was that having an abortion is as safe if not safer than that of pregnancy. I thought that was a big eye opener.”*
**Will today’s future session affect your future clinical practice? Describe why or why not.**
*“I feel more confident in what language to use when informing patients of their options//not assuming that a pregnancy is desired.”*
*“I think this session gave me great examples of what unbiased unassuming appropriate care looks like. I think we all may strive for that but sometimes it is difficult to articulate that in a way that is concise and direct. Overall, I definitely learned a lot of new skills”*

## Discussion

Together, the results demonstrate that a preclinical didactic lecture coupled with small-group discussions covering abortion care can be effectively implemented by a group of medical students with supervision from Family Planning faculty and institutional support. The initial goals of providing evidence-based information on abortion care and increasing student comfort with pregnancy options counseling were achieved, as evidenced by the significant improvement in correct answers for the post-session survey knowledge questions and the responses to the attitude statements. However, the retention of this knowledge over a time period was not assessed. The value of the session was supported by overall positive feedback from students, which is consistent with other recent surveys demonstrating medical student support for abortion education [8, 9].

The aim of our intervention was not to change students’ beliefs or attitudes regarding abortion. Rather, we aimed to provide evidence-based information surrounding a common medical procedure. However, we did consider that personal beliefs may influence how the session is received. Though attitudes on abortion are more complex than simply “pro-life” or “pro-choice,” participants in our study were more likely to consider themselves closer to “pro-choice” than “pro-life.” We acknowledge that presenting to an audience who largely supported abortion may have contributed to the positive feedback that we received. This session could be viewed and assessed differently in areas with more diverse views on abortion. Our finding was consistent with the results of a problem-based learning module at the University of Louisville, located in a state with more restrictive abortion legislation, which also proved to increase student knowledge of abortion care [10]. Regardless of personal beliefs, our presentation informed students on the science and clinically relevant aspects of abortions.

Given our promising results, this session is now a required part of the preclinical doctoring course for future cohorts of second-year medical students at our institution. In order to sustain this student-led model in the future, we integrated the responsibility of coordinating this lecture into an existing position within the obstetrics and gynecology student interest group. We plan to continue to invite faculty and residents to facilitate the small group discussions. We will consider presenting in person when university restrictions are lifted, although holding the session via Zoom allowed for increased faculty and resident participation. Further efforts will be centered on integrating pregnancy options counseling into other aspects of pre-clinical education, including standardized patient encounters in clinical skills sessions and assessing student ability to lead pregnancy options counseling.

This session provides one part of a solution for improving abortion education in the setting of the overturn of Roe v. Wade. The American College of Obstetrics and Gynecology supports the implementation of abortion education sessions throughout medical school [6]. Further, abortion training is a required component of obstetrics and gynecology residency programs accredited by the Accreditation Council for Graduate Medical Education [11]. Despite support from these institutional bodies, legislation severely restricts abortion training, whether by bans on who can perform the procedure, the practice setting in which it can occur, or the volume available for learning correct and safe care. Even with the original ruling on Roe v. Wade intact, 27% of surveyed obstetrics and gynecology residency programs cited state laws as restrictions to abortion training [12]. With the overturning of Roe v. Wade, political interventions in reproductive health has already further limited education and essential training on abortion in the clinical space, especially in states already implementing restrictive legislature [5]. Given the politicization of abortion, limitations in accessing abortion training may become a reality in other parts of the world where it is currently legal. In countries where abortion is banned, abortions continue to occur with deadly complications due to unsafe conditions and untrained physicians [13]. Further research will be needed to analyze changes to abortion education both in the preclinical and clinical settings in this new political environment.

## Conclusions

Currently, abortions are one of the most common procedures performed in the United States (US) with one in four women undergoing abortion by the age of 45 [14]. Despite the predicted decrease in exposure to clinical abortion education, students will likely eventually care for patients who need or have had abortion care. Addressing the issue of decreased educational opportunities and its consequences will require many approaches to effectuate change. We demonstrate one such successful approach: implementing a student-led formal education on abortion before students enter clinical spaces. Introducing the topic earlier in medical education will prepare more students and future providers to support patients who require this form of healthcare. We hope that this will inspire students in more restrictive states to advocate for this fundamental part of their education.

## Electronic supplementary material

Below is the link to the electronic supplementary material.

Supplementary Material 1

Supplementary Material 2

Supplementary Material 3

## Abbreviations

UCI: University of California Irvine
