# Supplementary material for: Medical student-led implementation of preclinical abortion didactic session at a California medical school
Source: BMC Med Educ. 2023 Jun 14;23:440. doi: 10.1186/s12909-023-04395-x (PMC10266871; doi:10.1186/s12909-023-04395-x)
Supplement: Supplementary file 1 — Supplementary Material 1 [file 12909_2023_4395_MOESM1_ESM.pdf]

Pre-Test

(if you haven't already)

Abortion Care 101:

What Every Physician Needs to Know

Margot Barker BS, Anna Cardall BS, Irene Masini BS, Yamini Patibandla BS, Katherine Rosecrance BS & Jasmine Patel, MD MSc

Clinical Foundations II
August 31st, 2021

Pre-Test

(if you haven't already)

Overview

1. Epidemiology & Safety
2. Duty of the Physician to the Patient
3. Pregnancy Options Counseling
4. Types of Abortion
5. Current Abortion Landscape: Federal & State
6. Resources
7. Case Discussion

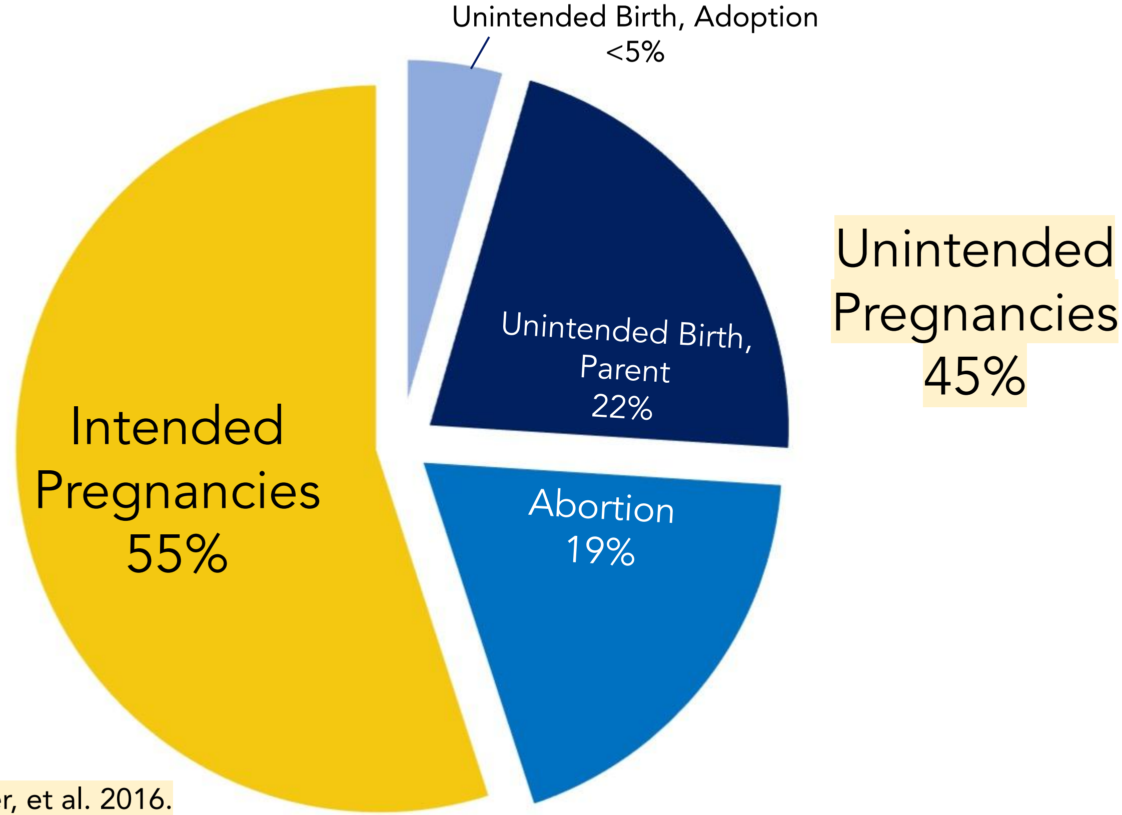

Finer, et al. 2016.

Abortion Epidemiology

- 1 in 4 people with uteri will have had an abortion by age 45[1]
- 51% of abortion patients used a contraceptive method in the month they became pregnant (2014)[1]
- 88% of abortions occur in first 12 weeks[2]

WHEN WOMEN HAVE ABORTIONS

In 2016, two-thirds of abortions occurred at eight weeks of pregnancy or earlier, and 88% occurred in the first 12 weeks.

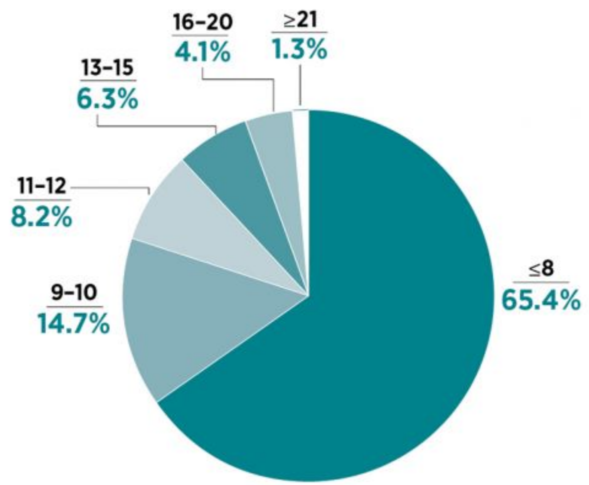

www.guttmacher.org

U.S. Abortion Patients

INCOME
75% poor or low income

RELIGION
62% religiously affiliated

FAMILY SIZE
59% already have a child

RACE
39% White

28% Black

25% Hispanic

6% Asian/Pacific Islander

3% Other

AGE
60% are in their 20s (only 12% are teens, of which 4% are minors)

Image from Guttmacher Institute 2016

Race & Abortion Care in the United States

- No race/ethnicity made up majority of abortion patients (2014)[3]
- Abortion rates across race vary widely: 27.1 for Black patients to 10.0 for white patients[3]
- Abortion rates are decreasing, but with a steeper decline for patients of color[3]
  - Clinic closures
  - Reduced access to care
  - Targeted restrictions

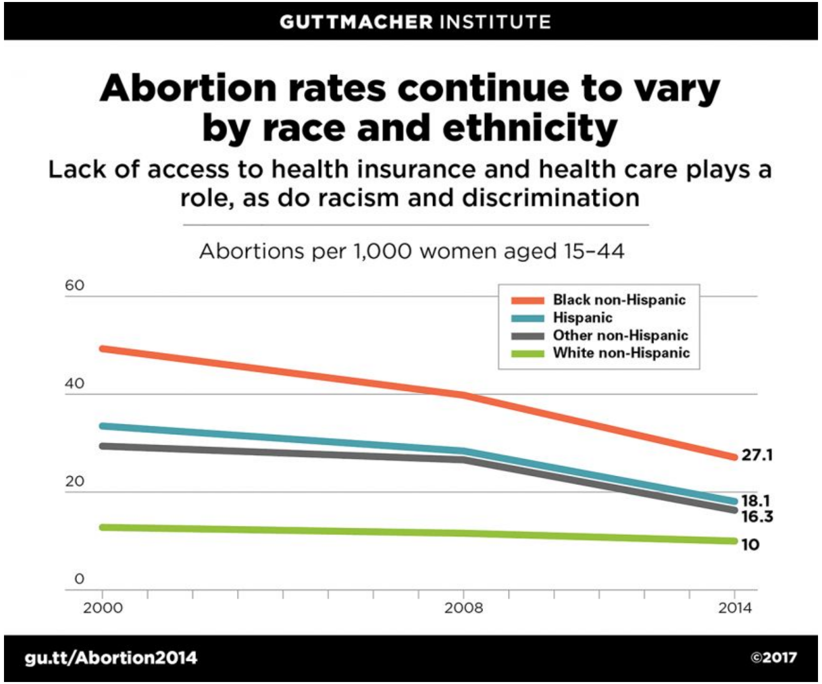

Safety of Abortion Care

Abortions are safe when performed in a safe environment

- Major complications (*hemorrhage, infection*) arose in less than 0.25% of abortion cases[4]
- Risk of death is 14x higher with childbirth[5]
- No future risk to fertility or increased risk for cancer
- Turnaway study: Patients "turned away" from abortion care were more likely to have:
  - 4x increased chance of living below federal poverty line
  - Increased anxiety and decreased self-esteem within 5 years
  - Higher chances of staying with abusive partners

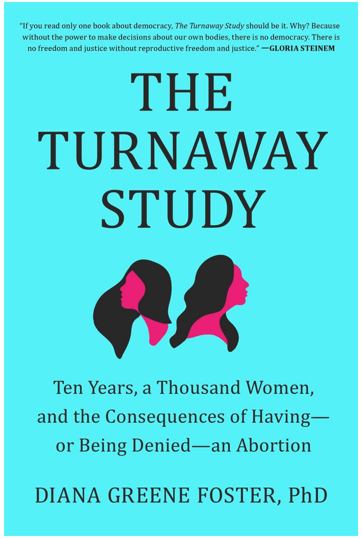

Duty of the Physician to the Patient

As the position of American College of Obstetricians and Gynecologists (ACOG) states:

- Induced abortion is an essential component of reproductive healthcare
- Healthcare providers should not seek to impose their personal beliefs on their patients, nor should they allow their personal beliefs to compromise patient health or access to care
- Providers have an ethical obligation to provide accurate information to their patients so that the patient may make their own fully informed decision; there is no room for provider bias in this conversation

But What If I Don’t Support Abortion?

- Healthcare providers have the option for conscientious refusal (refusing to provide care due to a conflict of conscience)[9]
  - SHOULD NOT impose religious/moral beliefs on patients, negatively impact patient health, be based on misinformation, or create/reinforce racial or socioeconomic inequalities[9]
- Physicians are still duty-bound to provide medically accurate and unbiased information to their patients and MUST refer patients in a timely manner to other providers[9]
  - Cannot impede patient care with conscientious refusal[9]
- Emergency situations: physicians have an obligation to provide medically indicated and requested care[9]

Patient-Centered Pregnancy Options Counseling

Discussion goals: provide effective, patient-centered, non-directive counseling to your patient.

You should address:

- Abortion
- Adoption
- Parenting

Every discussion will be different, there is no "one-size-fits-all" approach. What is right for one patient may not work for another.

Bottom line: listen to your patients and work with their individual situations.

Discussion Framework

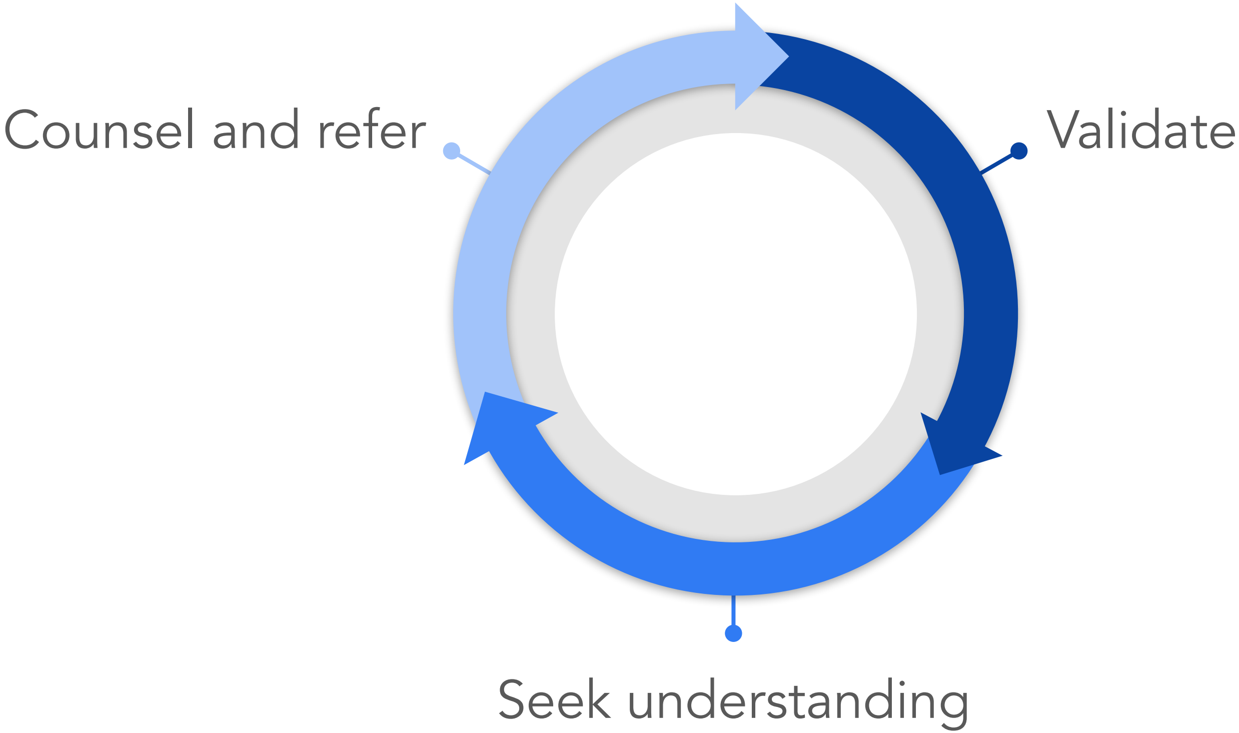

Role-Play

- Imagine you are working in the ER and are seeing a patient for nausea.
- You just found out she has a positive pregnancy test and go to tell her…

Types of Abortion

Medication Abortion (MAB)

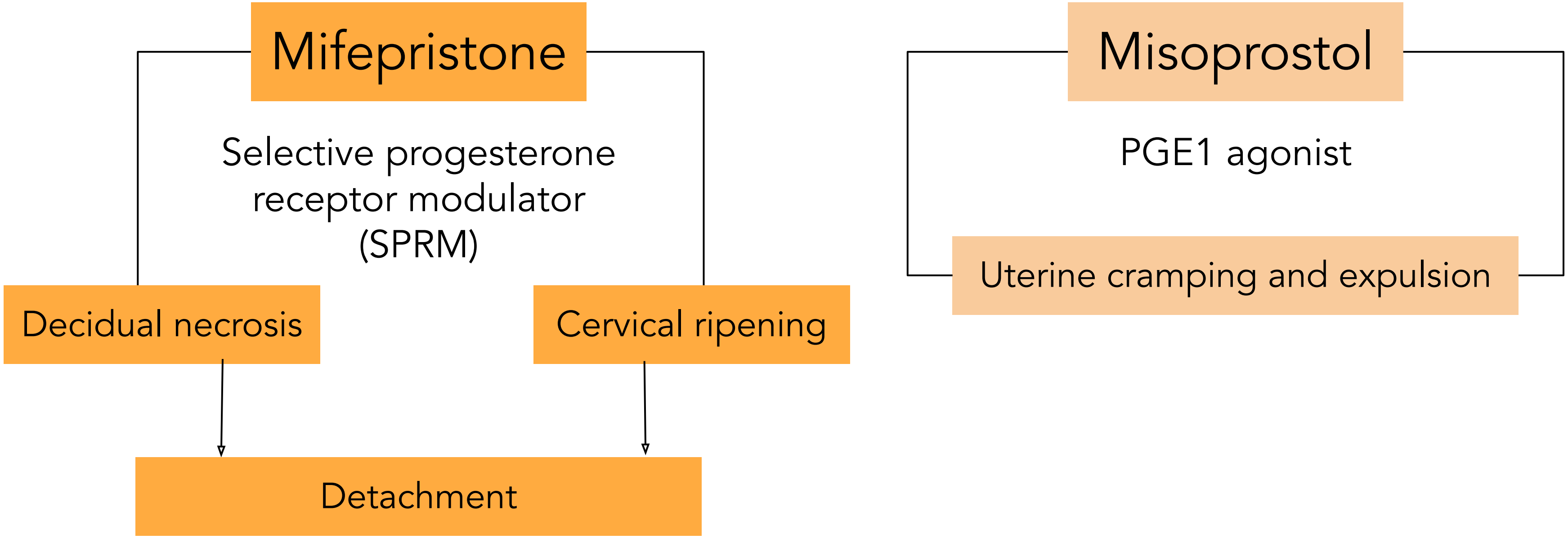

Medication Abortion

- Mifepristone + misoprostol is FDA approved for up to 70 days (≤10wks)
- Evidence for up to 77 days (≤11wks)
- Mifepristone + misoprostol have synergistic effects
- Patients are managed on an outpatient basis

Effectiveness by Gestational Age 200 mg Mifepristone and 800 mcg Misoprostol* (Interval 24-48 hours apart)[11]	
Gestational Age	Effectiveness
≤49 days	98.1%
50-56 days	96.8%
57-63 days	94.7%
64-70 days	92.7%

*buccal administration

Medication abortion "reversal" is not supported by science.

2012 – Delgado & Davenport: case series, n=7 variable prog. doses and unknown mife dose, 0 controls: 4 FT, 2 Ab, 1 N/A

2015 – Grossman et al: sys. review, no evidence that prog after mife $\not>$ expectant mgmt. for cont. pregnancy

2017 – Garratt & Turner: 3 cases of reversal in Australia: 2 FT, 1 Ab

2018 – Grossman & White: NEJM perspective, summarizing lack of evidence on the treatment's safety, effectiveness

2018 – Delgado et. al: retrospective analysis 754 cases of reversal tx: 257/547 (47%) live births

2020 – Creinin et al: RCT, n=12 oral prog after mife Study stopped early for safety concerns, no effect of prog tx found

Source: Advancing New Standards in Reproductive Health, Issue Brief July 2020 "Abortion Pill 'Reversal': Where's the Evidence?"

Manual Vacuum Aspiration (MVA)

- Prep patient (abx & pain control)
- Antiseptic prep and nerve block at cervix
- Dilate cervix manually*
- Insert cannula and attach to syringe
- Suction and evacuate uterine contents
- Confirm by inspecting POC

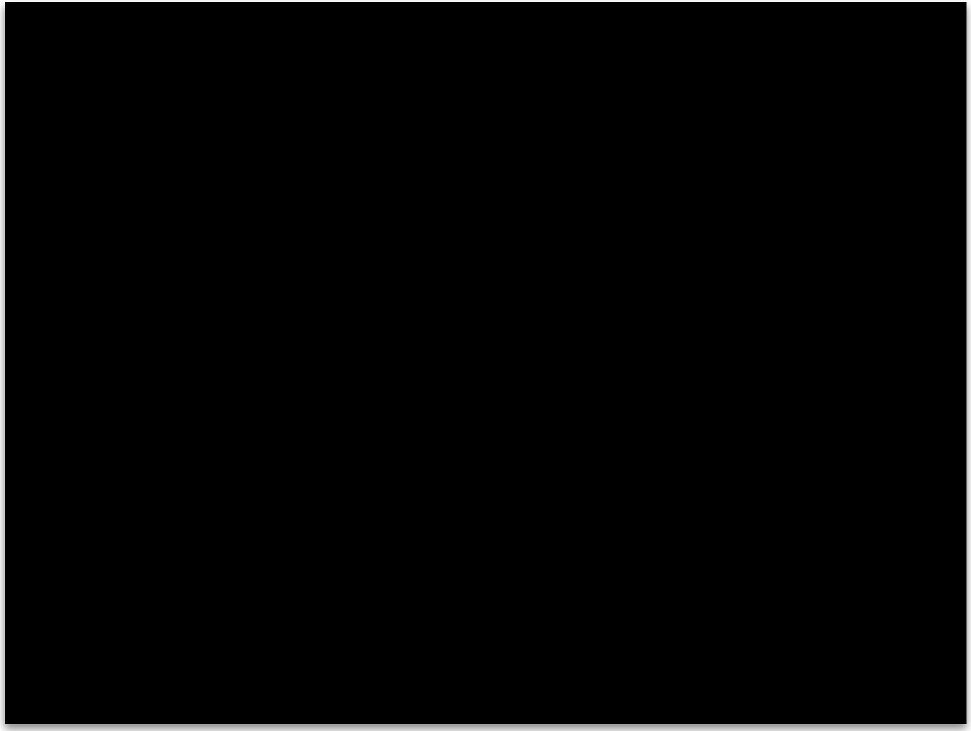

*cervix dilation dependent on gestational age
POC - products of conception

Dilation & Suction Curettage (D&C)

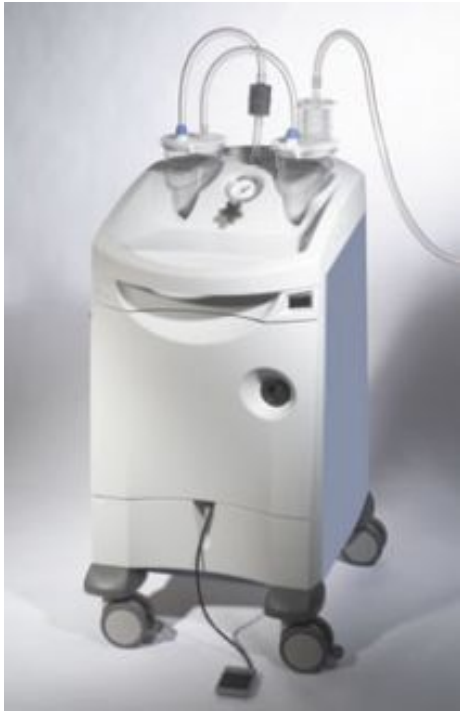

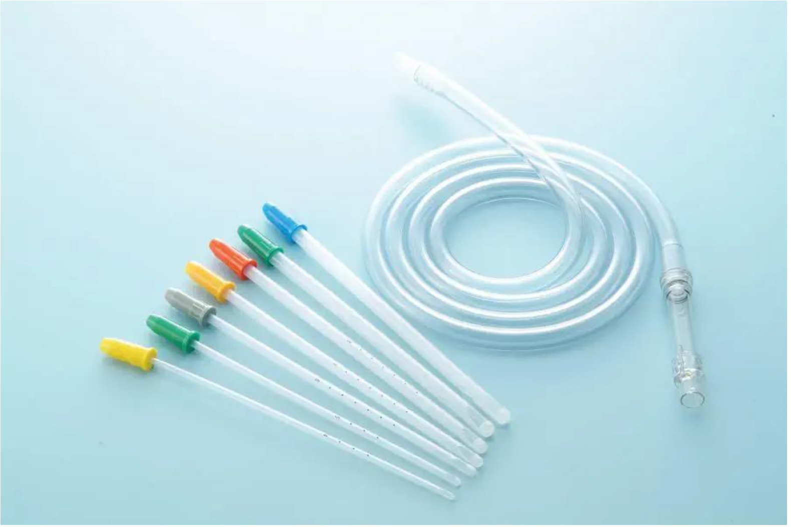

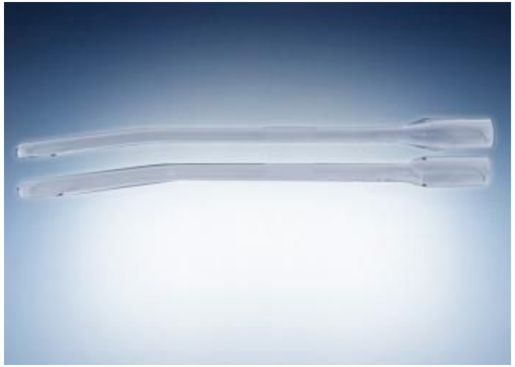

Left - electric pump
Center - flexible cannulas and long tubing
Right - rigid cannulas

Products of Conception (POC) ~9wk GA

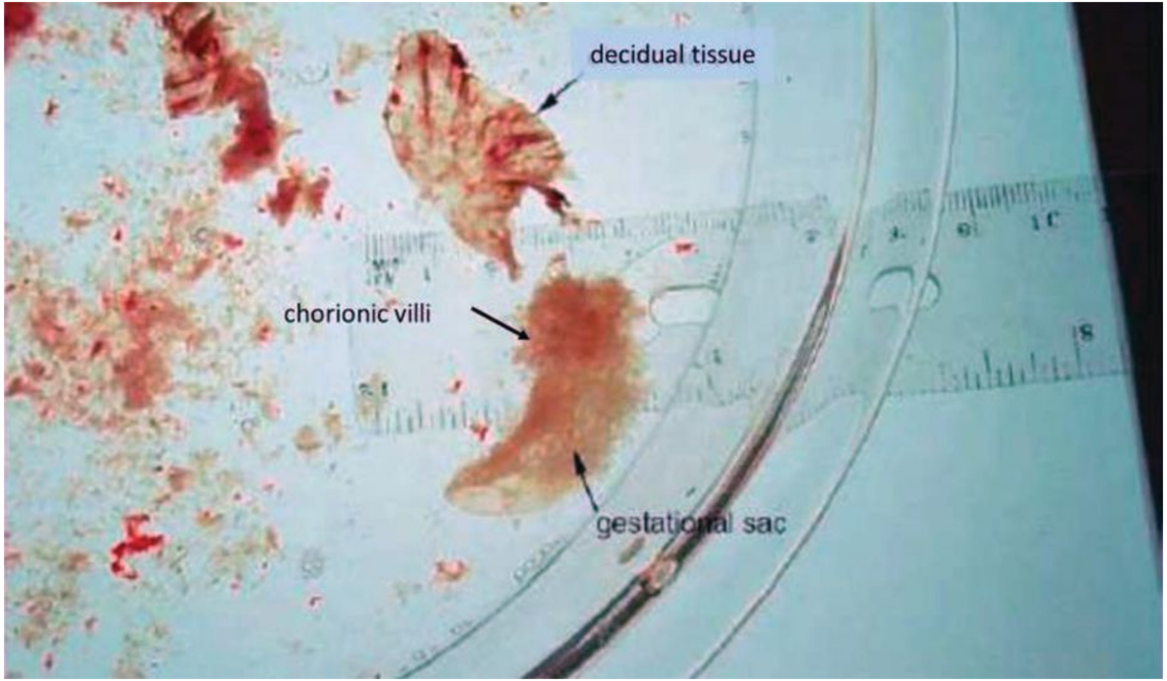

Dilation & Evacuation (D&E)

- 2nd trimester abortion (after week 14)
- 2 day procedure for cervical preparation and uterine evacuation
  - Cervical dilation with overnight osmotic, pharmacologic and/or mechanical dilators
  - Evacuation with suction, extraction forceps, curettage
  - Optional: induced fetal demise with digoxin or severed umbilical cord
- Typically performed up to 24 weeks

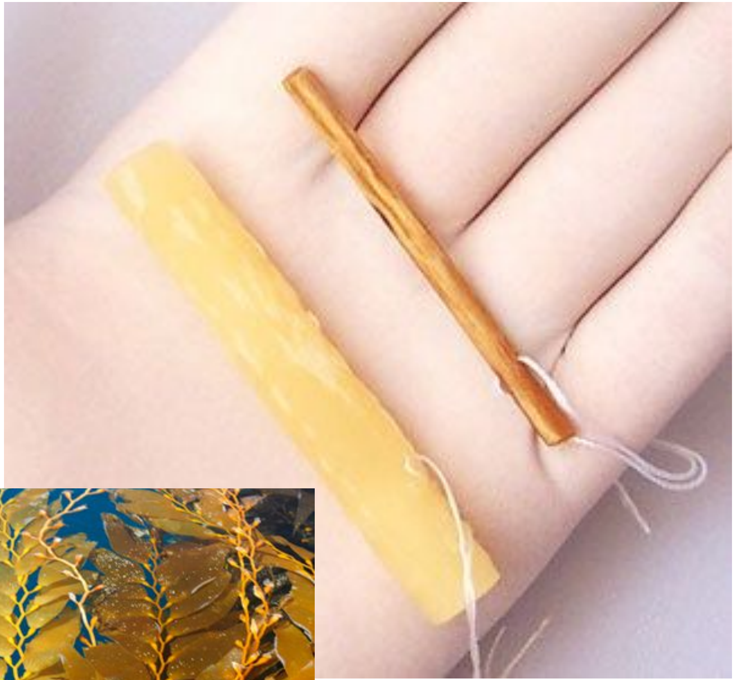

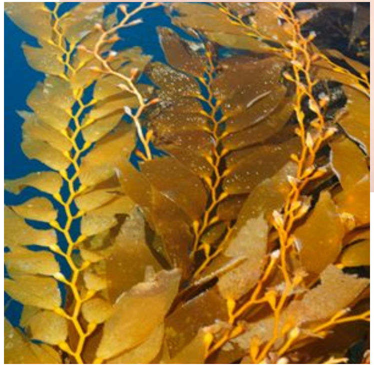

Induction of Labor (IOL)

- Induce labor and deliver the fetus with contractions and while patient is awake for entire process
  - Commonly performed for patients preferring more awareness of termination process
- Use of misoprostol alone, mifepristone+misoprostol, or oxytocin*
- May take as long as 24 hours, performed frequently in labor & delivery unit
- Possible dilation & curettage (D&C) for placenta removal

*oxytocin used if misoprostol not available

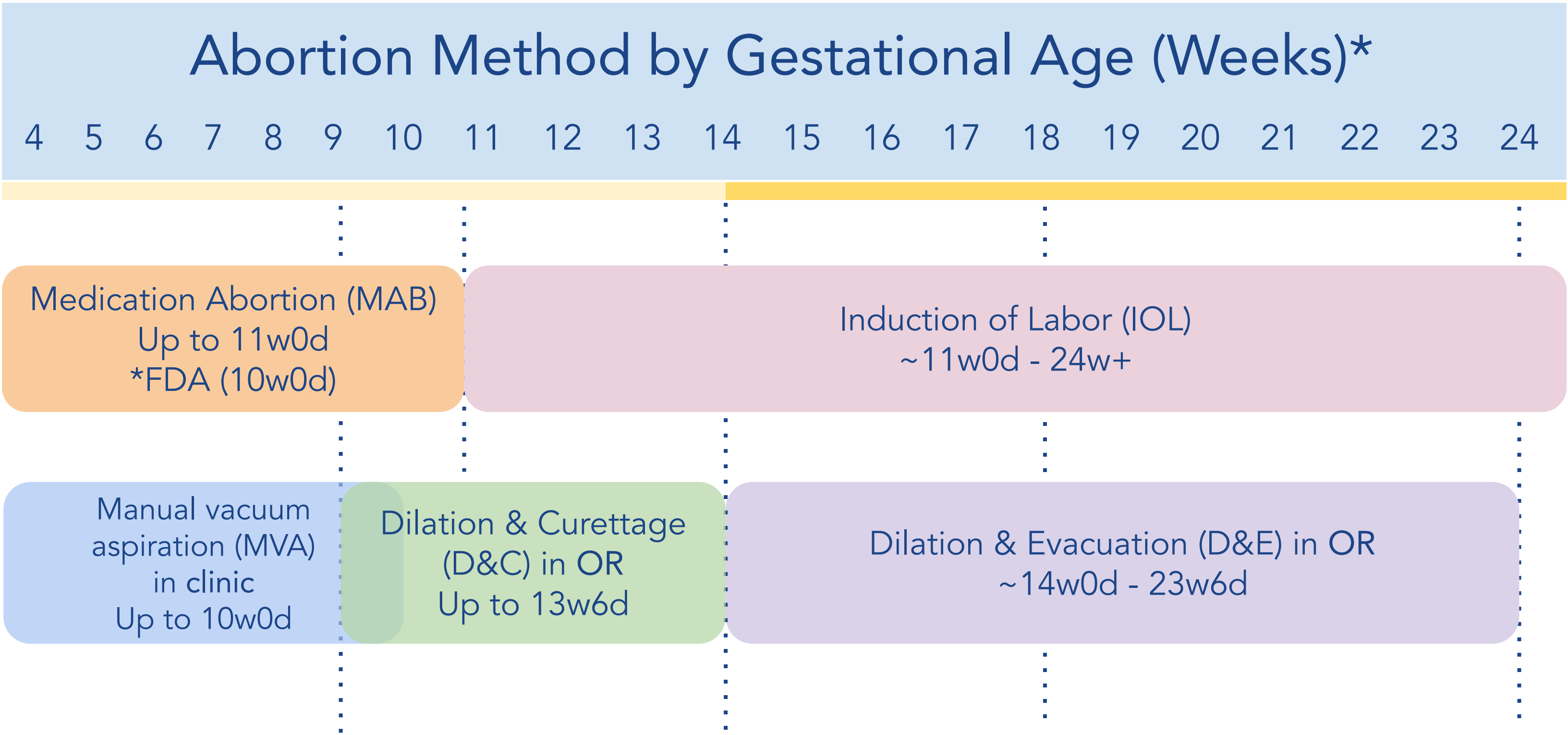

*can differ by sites, provider preferences

Landmark Supreme Court Cases & Funding

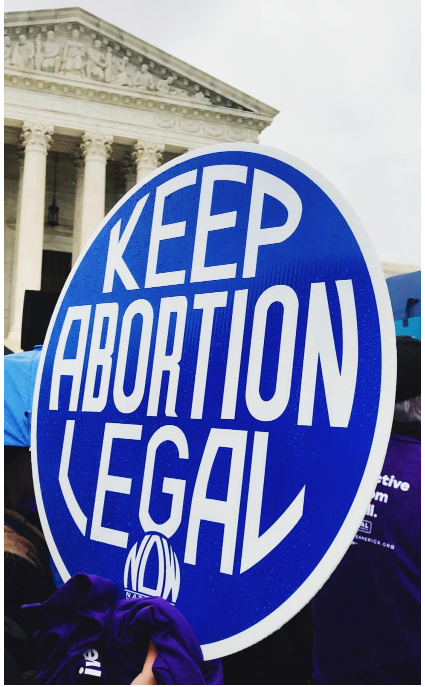

- Roe v. Wade (1973): "The constitutional right to privacy encompasses a woman's decision whether or not to terminate her pregnancy."
- Planned Parenthood v. Casey (1992): "State regulations … [cannot] place a 'substantial obstacle in the path of a woman seeking an abortion of a nonviable fetus.'"
- Hyde Amendment (1976): Ban of the use of federal funds on abortion services[12]

"In 2014, the majority of patients (53%) paid for their abortion out of pocket; [State] Medicaid was the second-most-common method of payment, used by 24% of patients." [13]

Restrictions to Roe

- Based on…
  - Fetus: GA, "viability," or trimester
  - Patient: Waiting periods, counseling, parental involvement
  - Provider: Targeted Restrictions to Abortion Providers (TRAP)[14]
    - Licensing restrictions
    - Width of hallways
    - Proximity to hospital
    - Admitting privileges
  - Technique: "Partial Birth" bans[15]

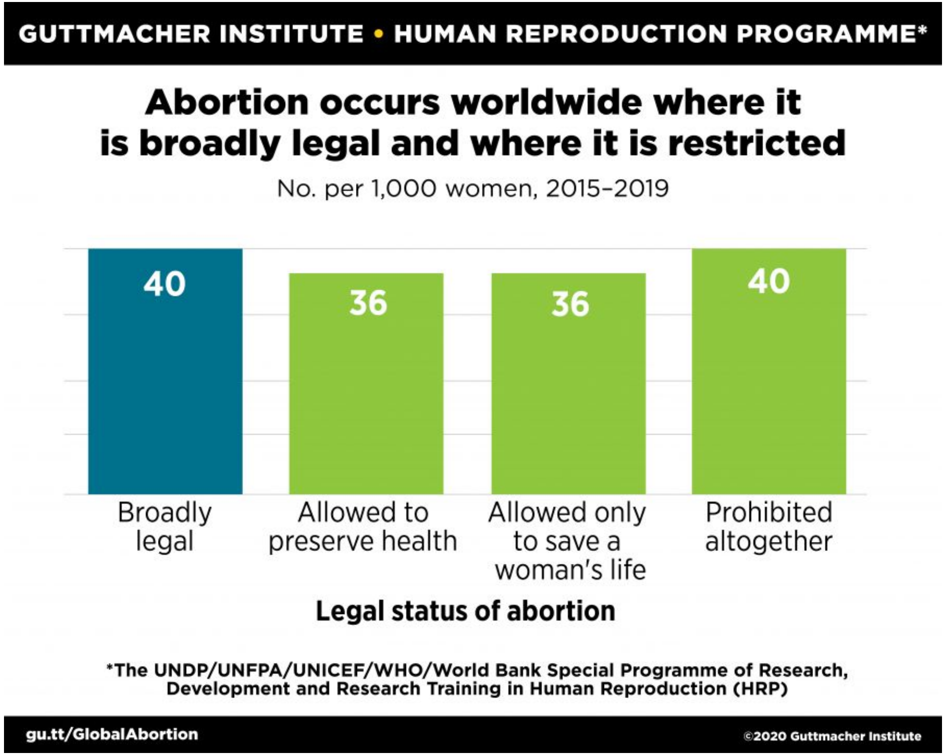

California Abortion Landscape

State Policies on Abortion Coverage in Medicaid, Private Insurance, and ACA Exchange Plans – 2020

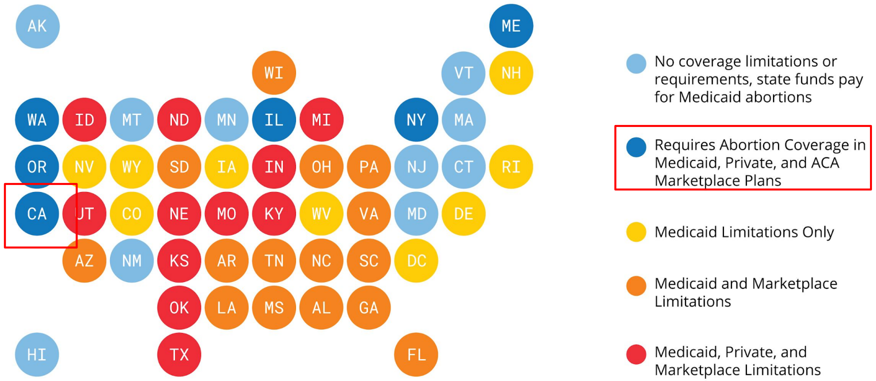

"How State Policies Shape Access to Abortion Coverage," Kaiser Family Foundation 2020.

CA Law: abortion is allowed prior to "viability," with the exception of endangerment to the patient's life[16]

Minors in CA: protected right to consent to abortion without parental consent

CA Funding: Medi-Cal covers abortion services (with state only funds) and private insurance companies are required to cover abortion services in state[17]

Abortion Resources in Orange County/California

(Images are linked to resources websites)

Women's Options Center

The UCI Health Women's Options Center (WOC) provides specialized care for women seeking contraception, pregnancy termination and management of miscarriage or perinatal loss.

Crisis Pregnancy Center Map

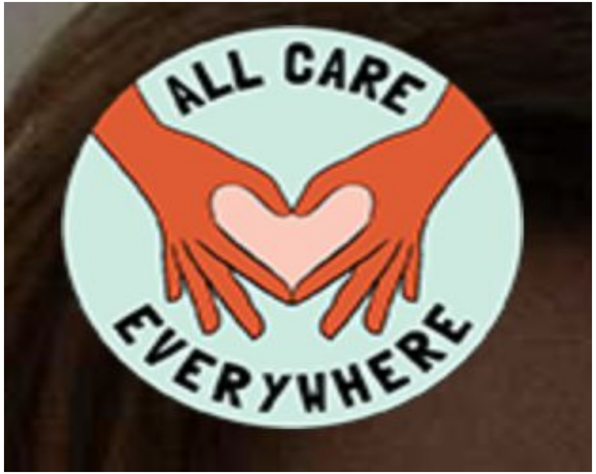

Planned Parenthood®
Orange and San Bernardino Counties

reproductive health access project

Case Discussion

Case Discussion

Heather, an unmarried 16-year-old high school student, becomes pregnant by her 17-year-old boyfriend of several months. She estimates she is 9 weeks pregnant and visits a doctor to ask about the options that she has. At this point in her pregnancy, what options can her doctor present to her?

1. Discuss with your group what Heather's pregnancy options are based on what you know so far.

2. What are the benefits and risks for each one of those options?

3. Assign someone to be the physician and someone else to be the patient. Practice patient-centered pregnancy options counseling.

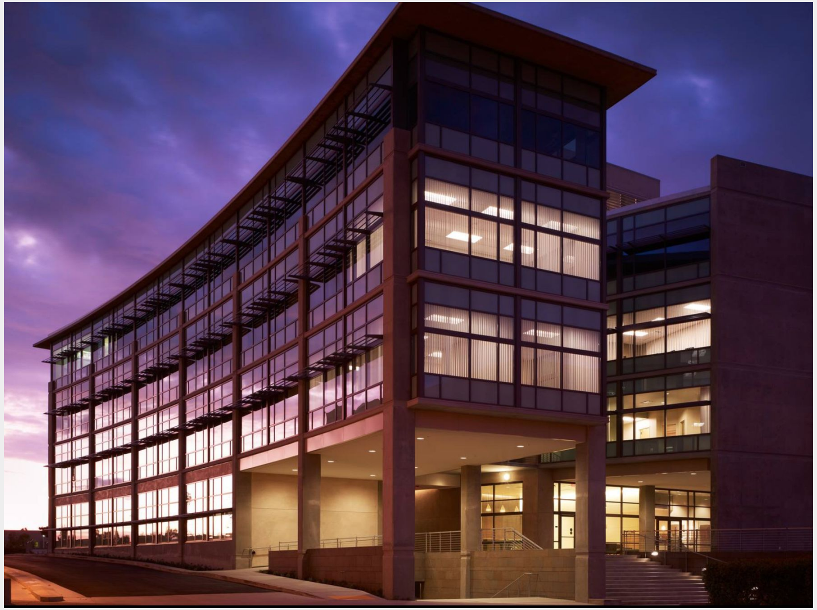

Case Discussion, continued..

Heather decides that she wants to get an abortion. Her parents have made it very clear that they would no longer allow her to live at home and would withdraw all financial support were she to become pregnant before marriage and have expressed strong opposition to abortion. Heather has always aspired to attend college and graduate school. Her family knows about her relationship with her boyfriend, but they are unaware of its sexual nature. When she goes to the doctor, they refuse to perform an abortion for Heather unless one of her parents provides consent for the procedure. Discuss the following questions with your group:

1. If Heather were living in California, what laws regarding medical care would apply to her?
    a. Would she be able to have an abortion without her parent's consent?
2. What are her options in terms of abortion at this time, having been pregnant for 9 weeks?
    a. What are the benefits and risks with these options? Which abortion method would be most appropriate for this patient?

Case Discussion, continued..

Heather is undecided about how to bring up her pregnancy to her parents, and time passes as she gathers the courage to ask. During this time, she has a busy month at school studying for her SATs. Finally, 5 weeks later, she asks her mom if she would be willing to provide consent for her abortion. Her mother is angry, but she finally agrees to consent to her abortion as long as Heather does not mention her pregnancy to others. With her signed consent, Heather returns to the doctor. At this point:

1. How have Heather's options changed in terms of the available methods of abortion?
    a. Weigh the risks and benefits of these options.
2. Had Heather not been able to access a safe method of abortion, what options would remain for Heather? How might these options affect Heather's physical health, emotional well-being, and long-term outcome?

Final Thoughts..

1. How do your responses to the above questions guide your support of Heather's decision-making authority free from parental consent?

2. If Heather's physician did not support abortion, what are the steps they could have to continue to abide with the law and fulfilled their duty as a physician?

3. How has this case informed your future career as a physician?

Post-test

References

1. Committee on Practice Bulletins—Gynecology; Society of Family Planning. Medication Abortion Up to 70 Days of Gestation: ACOG Practice Bulletin, Number 225. Obstet Gynecol. 2020 Oct;136(4):e31-e47. doi: 10.1097/AOG.0000000000004082. PMID: 32804884.
2. Finer LB and Zolna MR, Declines in unintended pregnancy in the United States, 2008–2011, *New England Journal of Medicine*, 2016, 374(9):843–852, doi:10.1056/NEJMsa1506575.
3. Rachel K. Jones and Jenna Jerman, 2017: Population Group Abortion Rates and Lifetime Incidence of Abortion: United States, 2008–2014. American Journal of Public Health 107, 1904_1909, https://doi.org/10.2105/AJPH.2017.304042
4. Induced abortions in the United States. Guttmacher Institute. https://www.guttmacher.org/fact-sheet/induced-abortion-united-states
5. Jones RK, Ingerick M and Jerman J, Differences in abortion service delivery in hostile, middle-ground and supportive states in 2014, *Women's Health Issues*, 2018, doi:10.1016/j.whi.2017.12.003.
6. Upadhyay UD, et al. Incidence of emergency department visits and complications after abortion. Obstet Gynecol 2015;125:175-83.
7. Raymond EG, Grimes DA. The comparative safety of legal induced abortion and childbirth in the United States. *Obstet Gynecol*. 2012;119(2 Pt 1):215-219. doi:10.1097/AOG.0b013e31823fe923
8. Abortion Policy | ACOG. https://www.acog.org/clinical-information/policy-and-position-statements/statements-of-policy/2020/abortion-policy.
9. The Limits of Conscientious Refusal in Reproductive Medicine | ACOG. https://www.acog.org/clinical/clinical-guidance/committee-opinion/articles/2007/11/the-limits-of-conscientious-refusal-in-reproductive-medicine.
10. Patient-Centered Pregnancy Options Counseling – RHEDI. https://rhedi.org/education/rhedi-curriculum/patient-centered-pregnancy-options-counseling/.
11. https://www.plannedparenthood.org/learn/abortion/the-abortion-pill
12. https://www.aclu.org/other/timeline-important-reproductive-freedom-cases-decided-supreme-court
13. Jerman J, Jones RK and Onda T, *Characteristics of U.S. Abortion Patients in 2014 and Changes Since 2008*, New York: Guttmacher Institute, 2016, https://www.guttmacher.org/report/characteristics-us-abortion-patients-2014.
14. https://www.guttmacher.org/state-policy/explore/targeted-regulation-abortion-providers
15. https://www.guttmacher.org/state-policy/explore/bans-specific-abortion-methods-used-after-first-trimester
16. https://www.prochoiceamerica.org/state-law/california/
17. https://healthlaw.org/storage/documents/NHeLP-CAAbortionCoverageFactSheet-Web.pdf
18. https://papayaworkshop.org/workshop-video/anesthesia-dilation-and-aspiration/
19. https://obgyn.onlinelibrary.wiley.com/doi/pdf/10.1111/tog.12072
20. https://www.innovating-education.org/2018/10/rhedi-patient-centered-pregnancy-options-counseling/
21. https://www.ansirh.org/sites/default/files/publications/files/so-called_medication_abortion_reversal_7-14-2020_1.pdf
22. https://www.acog.org/advocacy/facts-are-important/medication-abortion-reversal-is-not-supported-by-science
23. https://pubmed.ncbi.nlm.nih.gov/31809439/
24. https://prochoice.org/providers/quality-standards/
25.

Supplementary Figure 1:
Breakdown of patients having abortion by religion

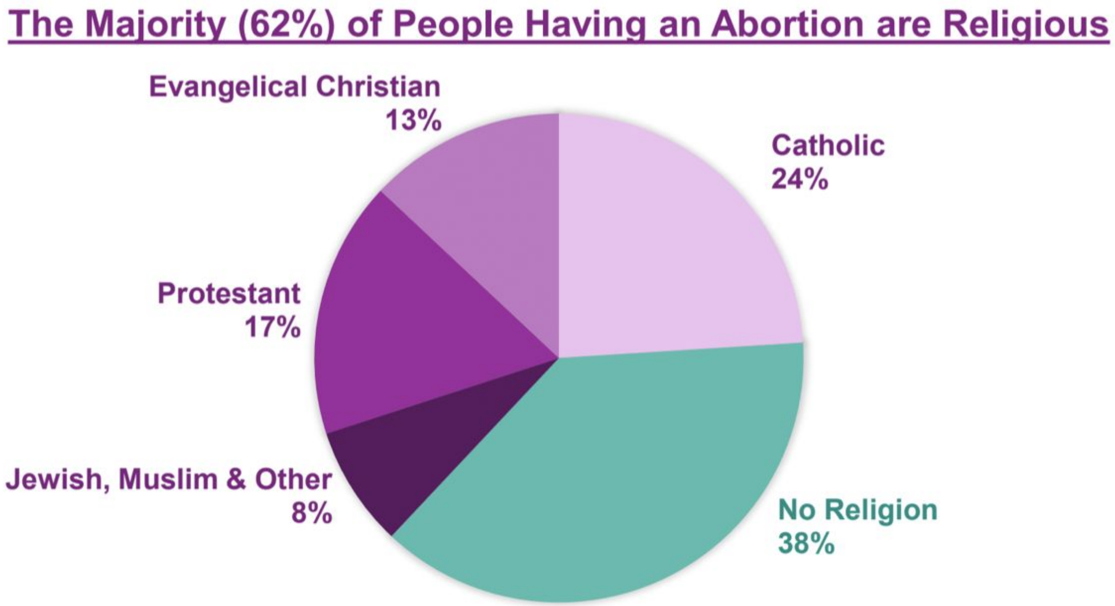

Guttmacher (2016)

Supplementary Figure 2:

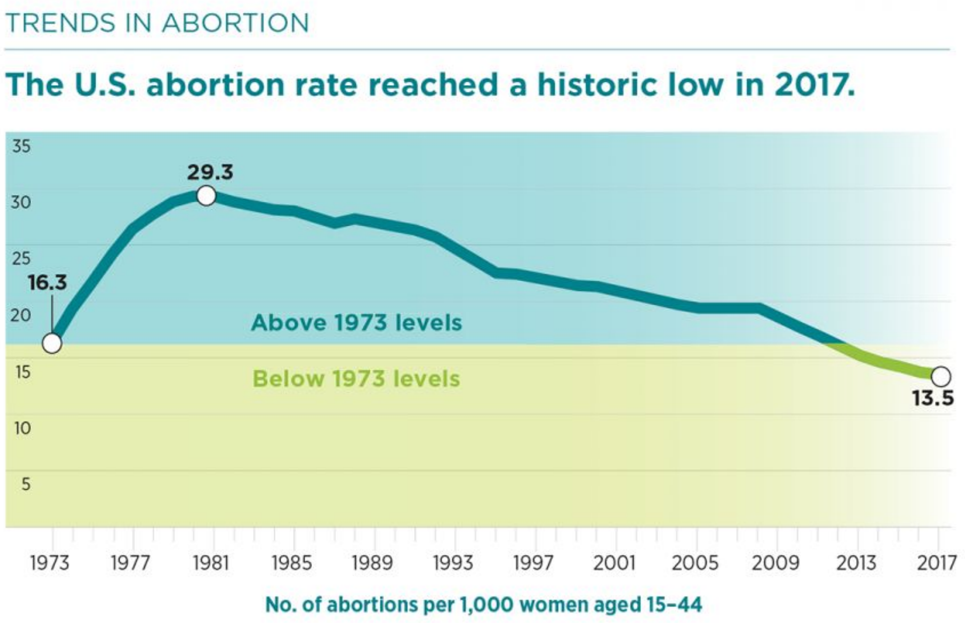

www.guttmacher.org

Supplementary Figure 3:

Table 2. Outcome by Gestational Age After Mifepristone 200 mg and Misoprostol for Outpatient Medication Abortion

	Misoprostol Dose	Interval Between Mifepristone and Misoprostol (h)	Gestational Age			
			≤49 days	50–56 days	57–63 days	64–70 days
Complete abortion	800 micrograms buccally[*]	24–48	98.1%	96.8%	94.7%	92.7%
	800 micrograms vaginally[†‡§‖¶‡‡]	24–72	98.3–99.7%	95.3–98.6%	95.1–98.3%	94.9%
	800 micrograms vaginally[§]	6–8	97.1%	94.2%	95.2%	N/A
	800 micrograms vaginally[‖¶]	0–0.25	95.5–95.7%	93.7–94.3%	91.6–95.3%	N/A
	400 micrograms sublingually[#**]	24–48	95.4%	N/A	94.8%	91.9%
Ongoing pregnancy	800 micrograms buccally[*]	24–48	0.3%	0.8%	2.0%	3.1%
	800 micrograms vaginally[†‡§‖¶‡‡]	24–72	0–0.4%	0–1.2%	0–2.2%	3.4%
	800 micrograms vaginally[§]	6–8	0.4%	0	0.8%	N/A
	800 micrograms vaginally[‖¶]	0–0.25	1.4–2.3%	1.9–2.8%	1.6–5.0%	N/A
	400 micrograms sublingually[#**††]	24–48	N/A	N/A	1.8–3.5%	2.2%

Abbreviations: h, hours; N/A, not available.

[*] U.S. Food and Drug Administration. Mifeprex (mifepristone) information. Postmarket drug safety information for patients and providers. Silver Spring, MD: FDA; 2018. Available at: https://www.fda.gov/Drugs/DrugSafety/PostmarketDrugSafetyInformationforPatientsandProviders/ucm111323.htm. Retrieved March 3, 2020.

[†] Schaff EA, Eisinger SH, Stadalius LS, Franks P, Gore BZ, Poppema S. Low-dose mifepristone 200 mg and vaginal misoprostol for abortion. Contraception 1999;59:1–6.

[‡] Schaff EA, Fielding SL, Westhoff C. Randomized trial of oral versus vaginal misoprostol at one day after mifepristone for early medical abortion. Contraception 2001;64:81–5.

[§] Creinin MD, Fox MC, Teal S, Chen A, Schaff EA, Meyn LA. A randomized comparison of misoprostol 6 to 8 hours versus 24 hours after mifepristone for abortion. MOD Study Trial Group. Obstet Gynecol 2004;103:851–9.

[‖] Creinin MD, Schreiber CA, Bednarek P, Lintu H, Wagner MS, Meyn LA. Mifepristone and misoprostol administered simultaneously versus 24 hours apart for abortion: a randomized controlled trial. Medical Abortion at the Same Time (MAST) Study Trial Group. Obstet Gynecol 2007;109:885–94.

[¶] Lohr PA, Starling JE, Scott JG, Aiken AR. Simultaneous compared with interval medical abortion regimens where home use is restricted [published erratum appears in Obstet Gynecol 2018;132:219]. Obstet Gynecol 2018;131:635–41.

[#] Raghavan S, Tsereteli T, Kamilov A, Kurbanbekova D, Yusupov D, Kasimova F, et al. Acceptability and feasibility of the use of 400 µg of sublingual misoprostol after mifepristone for medical abortion up to 63 days since the last menstrual period: evidence from Uzbekistan. Eur J Contracept Reprod Health Care 2013;18:104–11.

[**] Bracken H, Dabash R, Tsertsvadze G, Posohova S, Shah M, Hajri S, et al. A two-pill sublingual misoprostol outpatient regimen following mifepristone for medical abortion through 70 days' LMP: a prospective comparative open-label trial. Contraception 2014;89:181–6.

[††] von Hertzen H, Huong NT, Piaggio G, Bayalag M, Cabezas E, Fang AH, et al. Misoprostol dose and route after mifepristone for early medical abortion: a randomised controlled noninferiority trial. WHO Research Group on Postovulatory Methods of Fertility Regulation. BJOG 2010;117:1186–96.

[‡‡] Hsia JK, Lohr PA, Taylor J, Creinin MD. Medical abortion with mifepristone and vaginal misoprostol between 64 and 70 days' gestation. Contraception 2019;100:178–81.

Supplementary Figure 4:
Comparison of First Trimester Abortion Types

MABs	MVAs
Private (at home)	In office/clinic or OR
Patient controlled	Provider controlled
Unpredictable bleeding, might need MVA if incomplete	Infection, bleeding, perforation (rare!, <1%)
Must have follow-up within 7-14 days (ensure no retained POC w/ US, HCG)	One and done procedure! Can confirm no longer pregnant on same day
Building discomfort (up to 24 hours)	Very uncomfortable for shorter period of time (0-60 seconds) if only local anesthesia
