# Supplementary material for: Medical student-led implementation of preclinical abortion didactic session at a California medical school
Source: BMC Med Educ. 2023 Jun 14;23:440. doi: 10.1186/s12909-023-04395-x (PMC10266871; doi:10.1186/s12909-023-04395-x)
Supplement: Supplementary file 2 — Supplementary Material 2 [file 12909_2023_4395_MOESM2_ESM.pdf]

Abortion 101 Pre-survey

Please complete this survey prior to your attendance at the Abortion 101 session of Clinical Foundations 2. This survey is voluntary and anonymous. Results will be used to evaluate the effectiveness of the session and may be used for research purposes. Thank you for your participation.

Please create a 3 digit identifier for the purpose of matching your pre-survey with your post-survey. Use the first letter of your last name followed by the day of your birth (e.g. S19 for Mr. Smith born on the 19th).

____

What year in medical school are you in?

- ◯ MS1
- ◯ MS2
- ◯ MS3
- ◯ MS4

What year were you born?

____

What gender do you identify as?

- ☐ Man
- ☐ Non-binary
- ☐ Woman
- ☐ Not listed

Please indicate your gender identity:

____

What race/ethnicity do you identify as?

- ☐ American Indian or Alaska Native
- ☐ Asian
- ☐ Black
- ☐ Hispanic
- ☐ Middle Eastern
- ☐ Native Hawaiian or Pacific Islander
- ☐ White
- ☐ Not Listed

Please indicate your race:

____

Please indicate how you would currently qualify your personal beliefs in regards to abortion.

"Pro-life" ▭▭▭▭▭▭▭▭▭▭▭▭▭▭▭▭▭▭▭▭ "Pro-choice"

(Place a mark on the scale above)

In the United States, about what percentage of pregnancies end in abortion?

- ◯ < 1%
- ◯ 5%
- ◯ 10%
- ◯ 25%
- ◯ 50%

Having an abortion is more dangerous to the pregnant person than carrying the pregnancy to term.

- ◯ True
- ◯ False

All of the following are methods of abortion except:

- ◯ Medication abortion
- ◯ Vacuum aspiration
- ◯ Endometrial ablation
- ◯ Dilation and evacuation
- ◯ Induction of labor

	Strongly agree	Somewhat agree	Neither agree nor disagree	Somewhat disagree	Strongly disagree

I will encounter patients who have had or are considering having an abortion.	◯	◯	◯	◯	◯
I feel comfortable talking to my patients about abortion and pregnancy options.	◯	◯	◯	◯	◯
It is important to me that I am knowledgeable about abortion options and procedures.	◯	◯	◯	◯	◯
I believe that all physicians should be knowledgeable about abortion options and procedures.	◯	◯	◯	◯	◯
It is part of a physician's duty to provide comprehensive and accurate information about abortion to patients seeking this information.	◯	◯	◯	◯	◯
