# Supplementary material for: Medical student-led implementation of preclinical abortion didactic session at a California medical school
Source: BMC Med Educ. 2023 Jun 14;23:440. doi: 10.1186/s12909-023-04395-x (PMC10266871; doi:10.1186/s12909-023-04395-x)
Supplement: Supplementary file 3 — Supplementary Material 3 [file 12909_2023_4395_MOESM3_ESM.pdf]

Abortion 101 Post-survey

Please complete this survey following your attendance at the Abortion 101 session of Clinical Foundations 2. This survey is voluntary and anonymous. Results will be used to evaluate the effectiveness of the session and may be used for research purposes. Thank you for your participation.

Please enter the same 3 digit identifier you used for the pre-survey. Use the first letter of your last name followed by the day of your birth (e.g. S19 for Mr. Smith born on the 19th).

____

About what percentage of pregnancies end in abortion?

- ◯ < 1%
- ◯ 5%
- ◯ 10%
- ◯ 25%
- ◯ 50%

Having an abortion is more dangerous to the pregnant person than carrying the pregnancy to term.

- ◯ True
- ◯ False

All of the following are methods of abortion except:

- ◯ Medication abortion
- ◯ Vacuum aspiration
- ◯ Endometrial ablation
- ◯ Dilation and evacuation
- ◯ Induction of labor

	Strongly agree	Somewhat agree	Neither agree nor disagree	Somewhat disagree	Strongly disagree
I will encounter patients who have had or are considering having an abortion.	◯	◯	◯	◯	◯
I feel comfortable talking to my patients about abortion options.	◯	◯	◯	◯	◯
It is important to me that I am knowledgeable about abortion options.	◯	◯	◯	◯	◯
I believe that all physicians should be knowledgeable about abortion options.	◯	◯	◯	◯	◯
It is part of a physician's duty to provide comprehensive and accurate information about abortion to patients seeking this information.	◯	◯	◯	◯	◯
I believe today's session was valuable to my medical education.	◯	◯	◯	◯	◯

Did today's session change your views or attitudes regarding abortion?

- ◯ Yes
- ◯ No

Please describe why or why not.

____

Will today's session affect your future clinical practice?	◯ Yes<br>◯ No
Please describe why or why not.	__________________________________
What feedback do you have regarding the Abortion 101 session?	__________________________________
